# Relationship between phubbing and personality traits in Portuguese adults

**DOI:** 10.1192/j.eurpsy.2026.10862

**Published:** 2026-07-31

**Authors:** A. F. Oliveira, D. Antunes, B. R. Maia

**Affiliations:** 1 Faculty of Philosophy and Social Sciences; 2 Faculty of Philosophy and Social Sciences, Centre for Philosophical and Humanistic Studies, Universidade Católica Portuguesa, Braga, Portugal

## Abstract

**Introduction:**

Research suggests that personality traits such as neuroticism and extraversion can influence the tendency to engage in phubbing (ignoring other individuals in favor of one’s phone).

**Objectives:**

To explore the relationship between phubbing and neuroticism and extraversion in a sample of Portuguese adults.

**Methods:**

A sample composed by 304 subjects with a mean age of 27.04 (*SD* = 10.76; range = 18-69 years) completed a sociodemographic questionnaire, the Phubbing Scale (Phone Obsession and Communication Disturbance) and the revised Eysenck Personality Questionnaire (Extraversion and Neuroticism).

**Results:**

Significant and positive correlations were found between Neuroticism and Communication Disturbance (*r* = .17*, *p* =.003), and Phone Obssession (*r* = .18*, *p* = .002). Significant and negative correlations were found between Extraversion and Communication Disturbance (*r* =- .15**, *p* <.000). Females presented significantly differences in Phone Obsession levels (*M* = 13.29, *SD* = 3.16) versus males (*M* = 12.06, *SD* = 3.09), *t* (304) = 2.98, *p* <.001, with a moderate effect size (*d* =.39). Females also presented significantly differences in Neuroticism levels (*M* = 13.96, *SD* = 3.35) versus males (*M* = 11.16, *SD* = 5.66), *t* (264) = 3.79, *p* <.001, with a high effect size (*d* = 1.49).

**Conclusions:**

Our findings suggests that individuals with higher levels of neuroticism may be more prone to engaging in behaviors that disrupt relationship communicaition and create an over-reliance on their phones. Conversely, individuals with higher extraversion tend to engage less in phubbing behaviors. Additionally, females may experience more emotional instability and attachment to their phones. These findings underscore the importance of considering personality traits, especially neuroticism, and sex differences when examining the impact of mobile phone use on relationship dynamics and communication.

**Disclosure of Interest:**

A. Oliveira: None Declared, D. Antunes: None Declared, B. Maia Grant / Research support from: UIBD/00683/2020, FCT.

